# Efficient bone marrow irradiation and low uptake by non-haematological organs with an yttrium-90-anti-CD66 antibody prior to haematopoietic stem cell transplantation

**DOI:** 10.1038/s41409-024-02317-z

**Published:** 2024-06-12

**Authors:** Kim Orchard, Jonathan Langford, Matthew Guy, Gemma Lewis, Sofia Michopoulou, Margaret Cooper, Clint Zvavamwe, Deborah Richardson, Valerie Lewington

**Affiliations:** 1 Wessex Blood and Marrow Transplantation Programme, Department of Haematology, University Hospital Southampton NHS Foundation Trust, Southampton, UK; 2 NIHR/CRUK Experimental Cancer Medicine Centre, University of Southampton, Southampton, UK; 3 Department of Medical Physics, University Hospital Southampton NHS Foundation Trust, Southampton, UK; 4 Department of Nuclear Medicine, Bart’s and the London NHS Trust, London, UK; 5 Radiopharmacy, University Hospital Southampton NHS Foundation Trust, Southampton, UK; 6 School of Biomedical Engineering and Imaging Sciences, King’s College London, London, UK; 7 Present Address: PET Imaging Centre Facility, King’s College London, London, UK

**Keywords:** Phase I trials, Molecularly targeted therapy

## Abstract

We report the results of a Phase I radiation dose escalation study using an yttrium-90 (^90^Y) labelled anti-CD66 monoclonal antibody given with standard conditioning regimen for patients receiving haematopoietic stem cell transplants for myeloid leukaemia or myeloma. The ^90^Y-labelled anti-CD66 was infused prior to standard conditioning. In total, 30 patients entered the trial and 29 received ^90^Y-labelled mAb, at infused radiation activity levels of 5, 10, 25, or 37.5 megaBequerel (MBq)/kg lean body weight. A prerequisite for receiving the ^90^Y-labelled mAb was favourable dosimetry determined by single-photon emission computerised tomography (SPECT) dosimetry following administration of indium-111 (^111^In) anti-CD66. Estimated absorbed radiation doses delivered to the red marrow demonstrated a linear relationship with the infused activity of ^90^Y-labelled mAb. At the highest activity level of 37.5 MBq/kg, mean estimated radiation doses for red marrow, liver, spleen, kidneys and lungs were 24.6 ± 5.6 Gy, 5.8 ± 2.7 Gy, 19.1 ± 8.0 Gy, 2.1 ± 1.1 and 2.2 ± 0.9, respectively. All patients engrafted, treatment-related mortality 1-year post-transplant was zero. Toxicities were no greater than those anticipated for similar conditioning regimens without targeted radiation. The ability to substantially intensify conditioning prior to haematopoietic stem cell transplantation without increasing toxicity warrants further testing to determine efficacy. clinicaltrials.gov identifier: NCT01521611.

## Introduction

Autologous or allogeneic haematopoietic stem cell transplantation (HSCT) can improve outcomes for a wide range of haematological malignancies, however, the risks of treatment toxicity must be balanced against the risk of disease recurrence. In the allogeneic setting, total body irradiation (TBI) has been shown to reduce disease recurrence in acute myeloid leukaemia (AML) and chronic myeloid leukaemia (CML) in a dose-dependent manner [1–3]. The reduced relapse rate was, however, countered by corresponding increases in transplant-related mortality (TRM) at higher doses of radiation. Additionally, the doses of TBI used in full-intensity allogeneic transplantation protocols have excessive toxicity for older patients. Reduced-intensity conditioning protocols using lower radiation doses or avoiding TBI allow the extension of allogeneic transplantation to older patients and those with significant co-morbid conditions. The reduction of conditioning intensity results in lower TRMs, allowing engraftment and stable high donor chimerism but has an increased risk of disease recurrence, shown by several retrospective studies [4–8]. Essentially, the risks from the toxicity of the conditioning regimen are exchanged for increased risk of relapse resulting in similar overall survivals (OS) [9]. This has been confirmed in a large retrospective analysis of transplant outcomes [10] although randomised prospective trials have shown conflicting results, possibly due to differences in the details of conditioning used and the age limits applied [11–14]. A long-term follow-up study of a randomised trial showed a lower relapse risk in patients that received myeloablative conditioning while another long-term analysis showed no differences in TRM, relapse or OS [12, 13]. However, in patients aged 41–60 years, there was a significantly higher TRM with the higher TBI dose impacting on OS in these patients [12].

In the treatment of myeloma, the dose–response relationship led to the development of high-dose therapy and autologous stem cell transplantation, shown by several randomised trials to be of more benefit than chemotherapy alone and remains the recommended standard treatment [15–20]. Further dose escalation is limited by toxicity to non-haematopoietic organs. The use of tandem autologous transplantation allows dose intensification by performing two procedures temporally close, shown in some trials to result in improved outcomes [21–23] but at the expense of increased toxicity [24]. The addition of TBI to high-dose melphalan has been tested but was associated with increased TRM but no improvement in response rates or OS [25, 26].

An alternative to TBI that may reduce the incidence of complications while maintaining therapeutic intensity is targeted molecular radiotherapy (MRT) where radiation is targeted to antigens present on haematopoietic cells such as CD45 [27–31], CD33 [32, 33] and CD66 [34–36], principally using monoclonal antibodies as vector [37]. Several radioisotopes have been used clinically, mainly the beta particle emitting isotopes iodine-131 (^131^I), rhenium-188 (^188^Re) and ^90^Y [32] but also alpha-emitting isotopes such as bismuth-213 (^213^Bi) or astatine-211 (^211^As) [38], each radionuclide having advantages and disadvantages in the setting of HSCT. Although therapeutic doses of radiation can be delivered to haematopoietic tissues, a potential problem with the use of MRT has been the variable uptake and retention of the radiolabelled agent by non-target organs, particularly liver and kidneys, causing unwanted toxicities such as renal impairment and hepatic toxicity necessitating adjustment of the infused activity [39, 40], impacting on the potential benefit of the targeted radiation. The reasons for uptake by non-haematopoietic tissues are complex involving specific and non-specific binding or instability of the antibody-radioisotope construct in vivo. The variation of absorbed radiation in red marrow also complicates the assessment of disease response. Identifying optimal combinations of vectors and radioisotopes would allow the maximum potential benefit of targeted therapy to be achieved in all patients.

The cell surface antigen CD66 is an ideal target for MRT with isoforms of CD66 a, b, c and e present on cells of myeloid origin from promyelocytes through to mature neutrophils [39] and are also expressed by leukaemic blasts in 40% of patients with AML and in 80–100% of patients with B cell acute lymphoblastic leukaemia [40]. The isoform CD66a is expressed on plasma cells in the majority of cases of myeloma [41].

We report the results of a Phase I radiation dose escalation study using an ^90^Y-anti-CD66 in patients undergoing autologous or allogeneic HSCT for myeloma or high-risk myeloid leukaemia.

## Study design, patients, materials and methods

This was an open label, non-randomised Phase I study with four levels of infused ^90^Y-anti-CD66 radioactivity. Patients initially received ^111^In-anti-CD66 to determine the biodistribution and to derive organ dosimetry from whole body and SPECT gamma-camera images. Five patients were treated at each activity level with a planned expansion of the highest activity level to 15 patients if no dose-limiting toxicity (DLT) was demonstrated. The study was approved by the UK National Research Ethics Committee, the Administration of Radioactive Substances Advisory Committee and the Medicines and Healthcare Products and Regulatory Authority in accordance with the Declaration of Helsinki. Patients were recruited from referrals to the Wessex Blood and Marrow Transplantation Program for autologous or allogeneic HSCT as treatment for myeloma, AML or CML. Recruitment was dependent on the availability of nuclear medicine scanning time. Patients with AML had a high risk of relapse based on the presence of adverse cytogenetics, CR > 1 or secondary AML and were ineligible for full intensity conditioning due to age or co-morbid conditions. Patients with CML either had poor response to tyrosine kinase inhibitors or were in an accelerated phase. Exclusion criteria included hypocellular bone marrow with <10% red marrow content. AML patients in relapse were permitted with marrow blasts <20% of nucleated cells and peripheral blood leucocytes <30 × 10^9^/L.

Patients were informed of the investigational nature of the study and received verbal and written information before giving signed consent. The primary objective was to determine the safety profile of ^90^Y-anti-CD66 when included in standard transplant conditioning. Secondary objectives included determination of the maximum tolerated infused activity of ^90^Y-anti-CD66 as MBq/kg lean body weight, the radiation-related DLT in the context of standard autologous and allogeneic transplant conditioning schedules and development of a dosimetry model based on whole body and SPECT gamma imaging, blood clearance and thereby determine the pharmacokinetics of the radiolabelled antibody in vivo.

## Antibody description and radiolabelling

The anti-CD66 mAb is a murine IgG1, specific for a common epitope on CD66 isoforms a, b, c, e [42], International Non-proprietary Name, Besilesomab. The antibody was produced according to Good Manufacturing Practice and provided in a purified form by TheraPharm GmbH (Zug, Switzerland). Conjugation to isothiocyanato-2-aminobenzyl-3-methyl-diethylenetriamine-pentaacetic acid (SCN-2B3M-DTPA) [43] was performed on bulk batches and stored frozen in single use vials. For dosimetry the anti-CD66 was radiolabelled with ∼185 MBq of ^111^In and for therapy ^90^Y, the activity required based on treatment cohort and lean body weight (details in Supplementary Information).

## Dosimetry and pharmacokinetic studies

Patient-specific internal dosimetry was estimated using a methodology based on the Medical Internal Radiation Dose Committee and the EANM Dosimetry Committee [44, 45]. Cumulative activity in the blood was calculated from samples taken immediately at the end of infusion of 185 MBq ^111^In-anti-CD66 (*T* = 0) and at intervals up to day 7 post-infusion. Serial whole-body (WB) gamma-camera images were taken within 1 h of completion of infusion and at least three additional whole-body acquisitions between days 2 and 7 (details in Supplementary Information).

## Therapy with ^90^Y-labelled anti-CD66

Patients with a minimum twofold ratio between the calculated radiation dose to red marrow compared with the next highest non-haematopoietic organ, in all cases the liver, received therapy with ^90^Y-anti-CD66, administered on D-14 of the transplant schedule. Patients were discharged 2 h post-infusion. Patients receiving autologous HSCT were admitted on D-2 for melphalan 200 mg/m^2^. Patients undergoing allogeneic HSCT were admitted on day 5 post-infusion to start standard conditioning with fludarabine, melphalan and CAMPATH 1H, transplant conditioning index 2.5 [46] (detailed in Supplementary Information). Imaging and therapy were delivered as planned day case episodes.

## Patient characteristics

Thirty patients with myeloma, poor risk AML or CML were recruited into the study over a 5-year time period, mean age 54.4 years, range 20–68, 5 females and 25 males. Details of disease characteristics and number of prior therapies are summarised in Table 1. In patients with myeloma, disease stage was determined using the Durie–Salmon staging system which was in use at the time of this study [47].

**Table 1:** Patient characteristics.

Age at transplant yrs	Sex	Diagnosis	Prior treatment cycles	Disease status at transplant	Type of transplant
45	M	Myeloma	VAD × 6; oral melphalan × 1 cycle	PR	Autologous (1st)
55	M	Myeloma	VAD × 4	PR	Autologous (1st)
58	M	Myeloma	VAD × 4	PR	Autologous (1st)
65	F	Myeloma	VAD × 5	PR	Autologous (1st)
55	F	Myeloma	C-VAD × 6	CR	Autologous (1st)
48	M	Myeloma	C-VAD; autoHSCT; relapse, CTD salvage	PR	Autologous (2nd)
58	M	AML M6	DA, DA, MACE, MiDAC;	CR2	Allogeneic (sib)
			At relapse: DA, DA, HD ara-C		
20	F	AML M5a *t*(9; 11)	DA, DA, MiDAC	CR1	Allogeneic (sib)
43	M	Myeloma	C-VAD × 6	PR	Autologous (1st)
59	F	Myeloma	ZDEX; CTD	CR	Autologous (1st)
66	M	Myeloma	CTD × 6	PR	Autologous (1st)
56	M	Myeloma	VAD × 4; autoHSCT, relapse	PR	Allogeneic (sib)
56	M	Myeloma	VAD × 4; autoHSCT, relapse	CR	Allogeneic (sib)
53	M	Myeloma	C-VAD × 6	CR	Autologous (1st)
62	M	Myeloma	VAD × 6	PR	Autologous (1st)
65	M	Myeloma	CTD × 5	PR	Autologous (1st)
65	M	Myeloma	C-VAD × 6; Thal/Dex × 4	PR	Autologous (1st)
68	M	Myeloma	C-VAD × 5	PR	Autologous (1st)
60	M	Myeloma	ABCM × 6; VAD × 2; ZDEX × 4; CTD × 2	PR	Autologous (1st)
62	M	CML-CP	Imatinib	HR	Allogeneic (sib)
61	M	CML-AP	HU, IFN, Imatinib; autoHSCT; progression	CP2	Allogeneic (VUD)
61	M	AML M4, FLT3 mutation. Primary refractory.	DA; Salvage FLA-Ida × 2, HDAc	CR1	Allogeneic (VUD)
54	M	Myeloma, Lambda LCD	CTD × 4, autoHSCT, relapsed. Thal/Dex salvage	vgPR	Allogeneic (sib)
24	M	Secondary AML,	FLAG × 2, MACE × 1	CR1	Allogeneic (VUD)
		(previous HD. Mediastinal radiotherapy, autoHSCT)			
55	M	Secondary AML	DA × 2, HDAc, MiDAC	CR1	Allogeneic (sib)
45	M	Relapsed myeloma IgA kappa	VAD × 4, autoHSCT; relapse: CTD × 4; relapse 2, CTD × 3	CR3	Allogeneic (VUD)
57	M	Myeloma	CTD × 4, autoHSCT	vgPR	Allogeneic (sib)
		IgG kappa			
57	M	Relapsed AML M4	DA, DA, MACE, MiDAC. Relapse: FLAG × 2	CR2	Allogeneic (VUD)
36	F	AML M7	DA, DA, MiDAC	CR1	Allogeneic (sib)

*DA* daunorubicin + ara-C, *MACE* M-amsacrine + ara-C + etoposide, *MiDAC* mitoxanthrone + ara-C, *HDac* high dose ara-C, *C-VAD* cyclophosphamide + vincristine + adriamycin + dexamethasone, *VAD* vincristine + adriamycin + dexamethasone, *CTD* cyclophosphamide + thanlidomide + dexamethasone, *CR* complete remission, *PR* partial remission, *vgPR* very good partial remission. Myeloma disease response assessment were determined according to EBMT criteria; *VUD* volunteer unrelated donor, *HD* Hodgkin’s disease.

## Results

### ^111^In activity in whole blood

**Fig. 1:**
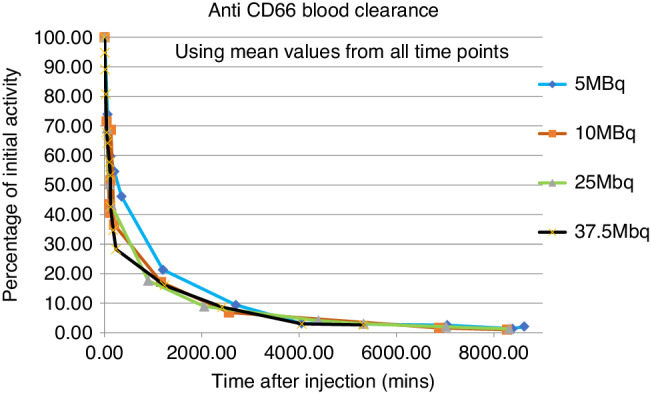
^111^In activity blood clearance curves. Mean blood ^111^In activity for each infused activity cohort in blood samples taken immediately at the end of infusion (*T*_0_) of ^111^In-anti-CD66 infusion and at timed intervals post-infusion. Initial blood ^111^In activity from *T*_0_ blood sample set as 100%; for subsequent samples, radioactivity expressed as a % of *T*_0_ activity, time in minutes post end of infusion.

**Fig. 2:**
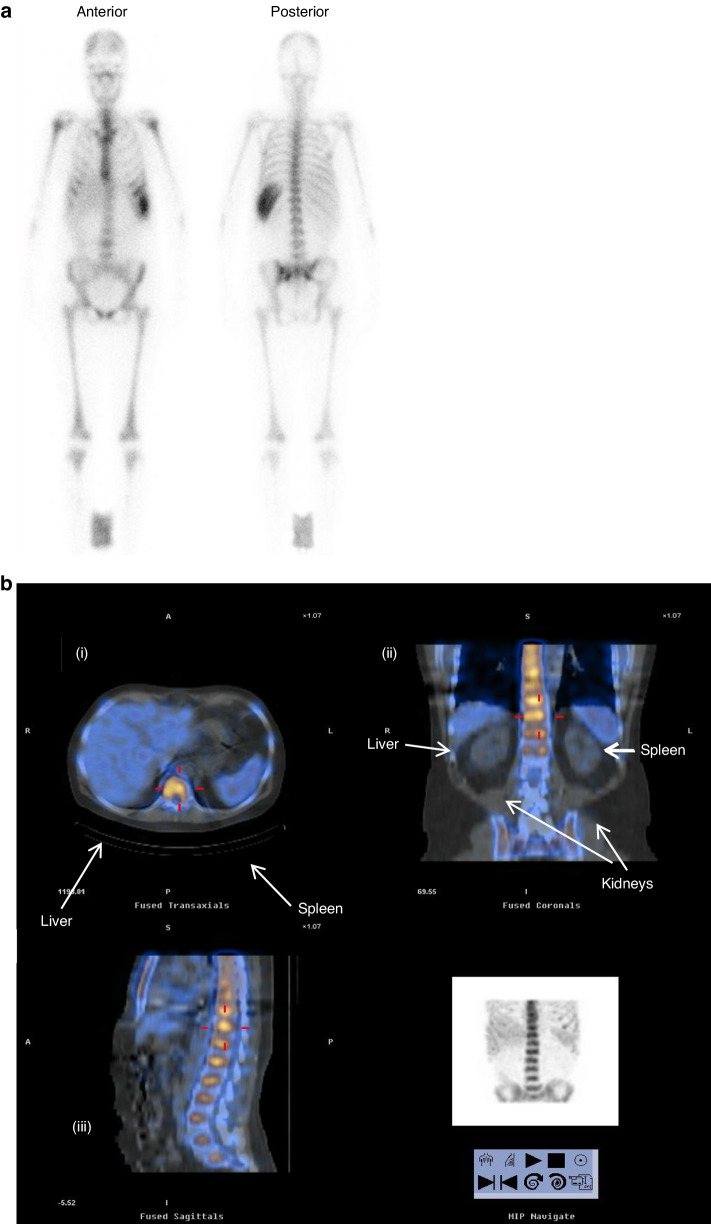
Planar and SPECT-CT gamma camera images post ^111^In-antiCD66 infusion. **a** Whole-body gamma-camera image showing distribution of ^111^In-anti-CD66 monoclonal antibody. **b** Superimposed SPECT gamma radiation (coloured) and CT images (grey scale). (i) Cross-section showing strong gamma radiation signal in L3 vertebral body (yellow), low in liver and spleen (blue) and undetectable in muscle, bowel. (ii) Coronal section image showing strong gamma radiation signal in vertebrae and pelvis (yellow), low in liver and spleen (blue) and very low in kidneys (grey scale image of CT). (iii) Sagittal image showing high gamma signal (yellow) in vertebral bodies, sternum and virtually none in other organs.

Blood ^111^In activity displayed similar biphasic retention in all patients, consistent with the sum of two exponential functions. In each patient, an initial rapid fall in blood ^111^In activity occurred within 2 h, followed by a slower decrease over 5–10 h (Fig. 1). After 24 h, the decrease in activity was markedly slower, approximating the physical half-life of the isotope and most of the radiation was located in the red marrow as indicated by whole-body planar gamma images (Fig. 2a) and SPECT(-CT) (Fig. 2b). From the early time-activity plots, the mean derived *T*_½_ alpha 2.06 ± 0.96 h (range 0.9–3.4), *T*_½_ beta 6.0 ± 3.2 h (range 4.0–9.0).

### Dosimetry

**Fig. 3:**
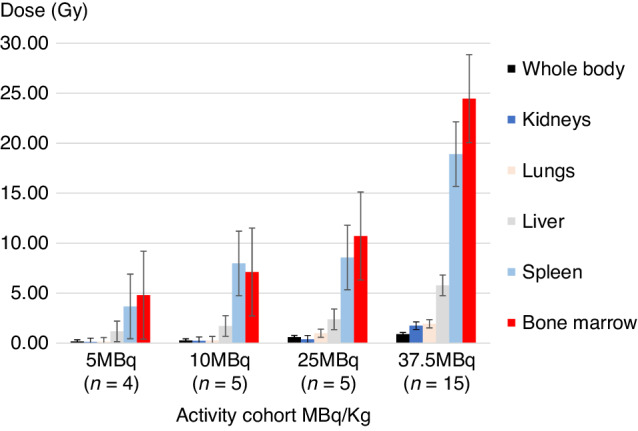
Histogram plots of specific organ dosimetry. The mean organ radiation dose determined from gamma-images for each of the four infused activity cohorts. Bars represent 1 standard deviation from the mean.

Thirty patients received ^111^In-anti-CD66. In the first cohort, one patient failed dosimetry with red marrow:liver ratio < 2:1 and was excluded from the study. Within the trial, 29 patients received ^90^Y-anti-CD66 at infused activity levels of 5, 10, 25 and 37.5 MBq ^90^Y per kilogram body weight, activity ranged from 229 to 2758 MBq, determined by patient weight and treatment cohort. In Table 2, the mean absorbed radiation dose to red marrow, spleen, liver, lungs and kidneys are expressed as milliGray per megaBequerel (mGy/MBq) of infused ^90^Y-anti-CD66 and the mean total organ doses are in Gy. The mean (±1 SD, range) estimated radiation dose delivered per unit infused radiation activity expressed as mGy/MBq were marrow 10.2 (±3.4, 6.5–16.2), liver 2.4 (±1.1, 1.4–5.6), spleen 9.0 (±3.9, 3.3–20.3), renal 0.6 (±0.4, 0.4–1.2), lungs 0.9 (±0.5, 0.5–1.8) and whole body 0.4 (±0.1, 0.2–0.6). The uptake of radiolabelled antibody by red marrow showed a high degree of consistency between patients. The mean red marrow to liver ratio was 4.3:1, estimated radiation doses to lung, kidneys, muscle and gut were considerably lower than to red marrow, shown graphically in Fig. 3. In most patients, the distribution of radiation was similar with marked uptake in the axial skeleton, ribs, sternum, pelvis, base of skull and proximal ends of the femur and humerus (Fig. 2a), consistent with red marrow distribution in adults.

**Table 2:** Organ dosimetry. Calculated absorbed radiation dose to critical organs expressed as mGy per infused MBq of ^90^Y-anti-CD66 and the mean estimated organ absorbed radiation doses in Gray (Gy) with standard deviation.

^*90*^*Y dose MBq/kg*	Mean Infused Activity [^90^Y] MBq	Bone marrow		Liver		Spleen		Renal		Pulmonary		Whole body	
		mGy/MBq	Gy	mGy/MBq	Gy	mGy/MBq	Gy	mGy/MBq	Gy	mGy/MBq	Gy	mGy/MBq	Gy
5	407 ± 132	*11.8* ± *1.5*	*4.8* ± *1.6*	*3.0* ± *0.8*	*1.2* ± *0.4*	*9.9* ± *4.9*	*3.7* ± *1.6*	*0.3* ± *0.1*	*0.1* ± *0.1*	*1.2* ± *1.1*	*0.5* ± *0.5*	*0.4* ± *0.1*	*0.2* ± *0.1*
10	781 ± 171	*10.3* ± *4.0*	*8.5* ± *1.3*	*2.4* ± *1.0*	*1.2* ± *0.3*	*11.0* ± *3.2*	*7.1* ± *3.3*	*0.4* ± *0.1*	*0.3* ± *0.1*	*0.4* ± *0.1*	*0.3* ± *0.1*	*0.4* ± *0.1*	*0.3* ± *0.0*
25	1452 ± 69	*10.7* ± *2.0*	*15.7* ± *3.5*	*2.4* ± *0.8*	*3.4* ± *1.0*	*8.5* ± *5.6*	*12.4* ± *8.2*	*0.5* ± *0.4*	*0.7* ± *0.5*	*1.1* ± *0.6*	*1.5* ± *0.9*	*0.4* ± *0.1*	*0.6* ± *0.1*
37.5	2196 ± 310	*11.3* ± *2.6*	*24.6* ± *5.6*	*2.7 ±1.3*	*5.8* ± *2.7*	*8.9* ± *3.9*	*19.1* ± *8.1*	*1.0* ± *0.6*	*2.1* ± *1.1*	*1.0* ± *0.5*	*2.2* ± *0.9*	*0.4* ± *0.1*	*0.9* ± *0.2*
Overall mean mGy/MBq		*10.1* ± *3.4*	–	*2.4* ± *1.1*	–	*9.0* ± *4.0*	–	*0.5* ± *0.4*	–	*0.7* ± *0.5*	–	*0.4* ± *0.1*	–

**Fig. 4:**
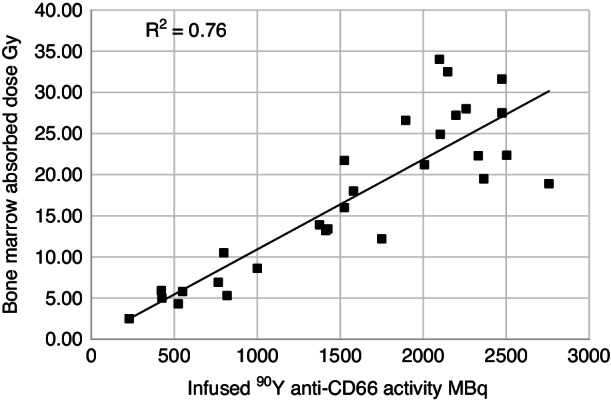
Infused ^90^Y-anti-CD66 activity in MBq and estimated radiation dose in Gy to bone marrow. The graph shows the calculated radiation dose in Gray (Gy) to the bone marrow plotted against the infused ^90^Y-anti-CD66 activity for each individual patient. For the ^90^Y-anti-CD66 activity levels used, the coefficient of determination, *R*^2^, was 0.76 indicating a close fit to a linear relationship between infused activity and bone marrow radiation dose. *R*^2^ is calculated using the Microsoft (MS) Excel software.

**Fig. 5:**
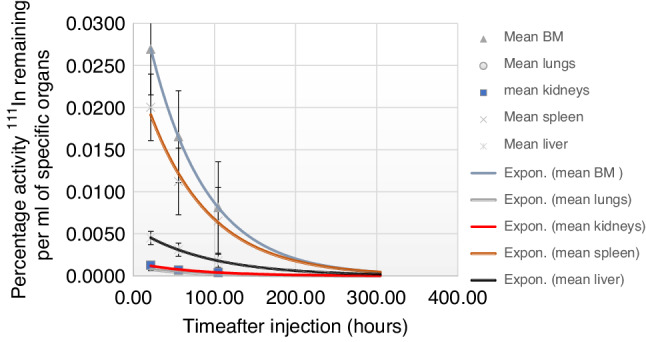
Time vs mean organ percentage activity based on initial ^111^In-anti-CD66 injected activity. Curve fitting achieved with the MS Excel software with a single exponential fit using gamma-camera-derived data. Lumbar vertebral body red marrow: *T*_eff_ = 48.14 ± 12.1 h. Liver: *T*_eff_ = 59.7 ± 12.9 h.

There was a linear relationship between the infused activity of ^90^Y in MBq and the absorbed dose to the bone marrow in Gy over all dose levels (Fig. 4) indicating that the uptake of labelled anti-CD66 mAb was consistent between patients for any given infused activity cohort. The targeted radiation remained localised within red marrow over the period of imaging. Additionally, red marrow time/activity curves for each patient showed a similar effective half-life (*T*_eff_) of 48.14 ± 12.1 h that was slightly shorter than the physical *t*_½_ for ^111^In (67 h) indicating good retention in the target organ (Fig. 5).

### Engraftment

Neutrophil recovery reached >0.5 × 10^9^/L at a median of 15 days (range 9–55) and platelet recovery >50 × 10^9^/L median 15 days (range 10–119) post-transplant. All patients had marrow aspirate and trephine biopsy performed at approximately day 100 post-transplantation, tri-lineage engraftment was seen in all cases. Importantly, no late graft failures occurred. Chimerism analysis in recipients of allogeneic transplants showed >97% donors in unselected peripheral blood cells by day 30 post-transplant in all patients.

### Safety profile

No immediate adverse effects were seen during the infusion of either ^111^In or ^90^Y-anti-CD66. Following ^111^In-anti-CD66 infusion, there were no changes in peripheral blood counts. Post ^90^Y-anti-CD66 infusion, all patients experienced grades 1–4 haematological toxicity, as indicated by a fall in peripheral blood counts, from days 8 to 12 post infusion. There was an increasing rate and depth of decline in counts with increasing total activity of ^90^Y-anti-CD66 infused. One patient experienced transient grade 1 gut toxicity with diarrhoea lasting 24 h post ^90^Y-anti-CD66 infusion, and another experienced transient grade 2 infection due to bacteraemia related to routine flushing of the Hickman line. The remaining patients experienced no toxicities, other than haematological, from the time of infusion up to the start of the conventional transplant conditioning. All episodes of grades 1–4 (WHO Toxicity Criteria) [48] nausea and vomiting were related to the infusion of high-dose melphalan, the severity recorded was no different to that seen in patients who had not received radiolabelled antibody (Table 3).

**Table 3:** Summary of all toxicities recorded, up to D + 100 post-transplantation.

Toxicity	Infused activity level MBq/kg			
	5 *n* = 4	10 *n* = 5	25 *n* = 5	37.5 *n* = 15
Gastrointestinal				
Bilirubin	1 grade 1	1 grade 4^a^	1 grade 1	4 grade 1
ALT	1 grade 1	3 grade 1	0	0
		1 grade 2		
GGT	1 grade 1	1 grade 1	1 grade 1	4 grade 1
		2 grade 2	1 grade 3	5 grade 2
				1 grade 3
ALP	1 grade 1	0	0	4 grade 1
Oral	2 grade 1	3 grade 1	1 grade 1	2 grade 1
	2 grade 3	2 grade 2	1 grade 2	2 grade 2
			2 grade 3	8 grade 3
			1 grade 4	3 grade 4
Diarrhoea	1 grade 1	3 grade 2	2 grade 2	4 grade 1
	3 grade 2	2 grade 3	3 grade 3	9 grade 2
				1 grade 4^b^
Constipation	0	0	0	2 grade 1
Nausea and Vomiting	3 grade 1	2 grade 1	1 grade 1	3 grade 1
	1 grade 2	3 grade 2	2 grade 2	4 grade 2
			1 grade 3	7 grade 3
Renal				
Urea	0	1 grade 1	2 grade 1	8 grade 1
Creatinine	0	1 grade 1	0	1 grade 1
Haematuria	0	0	0	5 grade 1
				1 grade 2^c^
Proteinuria	0	1 grade 1	0	7 grade 1
				2 grade 2^c^
Pulmonary	0	0	0	1 grade 1
				1 grade 1
				2 grade 2
Drug fever	0	0	0	3 grade 1
				7 grade 2
Allergic	0	0	0	0
Skin	0	0	0	2 grade 1
				2 grade 2
Infection	2 grade 1	3 grade 1	3 grade 1	2 grade 1
	1 grade 2		2 grade 2	12 grade 2
Cardiac				
Rhythm	0	1 grade 1^d^	0	4 grade 1^e^
Pericardial	0	0	0	0
Functional	0	0	0	0
Neurological				
Conscious	0	0	0	4 grade 1
Pain	3 grade 1	4 grade 1	3 grade 1	5 grade 1
	1 grade 2		1 grade 2	8 grade 2
				2 grade 3

^a^One patient experienced raised bilirubin; resolved on withdrawal of concurrent medication (norethisterone and itraconazole).

^b^Patient experienced grade 4 diarrhoea with PR bleeding – colonoscopy revealed a solitary rectal polyp that was removed. Episode unrelated to use of radiolabelled antibody.

^c^Patient experienced grade 2 proteinuria and grade 2 haematuria (microscopic) due to BK viral cystitis.

^d^One patient experienced transient asymptomatic atrial fibrillation and abnormal thyroid function tests indicative of hyperthyroidism (elevated T4, low TSH). Resolved after 1 month. Later developed hypothyroidism requiring thyroid replacement.

^e^Transient fever and associated tachycardia due to CAMPATH 1H infusion in allogeneic transplant recipients.

Organ toxicities recorded following completion of standard conditioning therapy and post-stem cell transplantation are summarised in Table 3 using WHO Toxicity Criteria. As predicted for patients receiving conditioning therapy for HSCT, all patients experienced grade ≥ 3 haematological toxicity. Gastrointestinal toxicity was comparable to that caused by conventional transplant conditioning, particularly that caused by high-dose melphalan. There was a trend for more patients to experience grade ≥ 3 oral mucositis at the higher radiation dose levels. The severity was within that expected for high-dose therapy alone, but because of the trend, this was considered to represent the DLT when used in conjunction with the dose of melphalan of 200 mg/m^2^. In patients undergoing allogeneic HSCT, the lower dose of melphalan, 140 mg/m^2^, caused less generalised mucositis and no clear DLT was seen. The use of enteral or parenteral nutrition was similar to that required in patients undergoing standard transplant conditioning without MRT. Two patients developed HAMA post autologous HSCT.

Transplant-related mortality was 0 at days 30, 100, 180 and 1-year post-transplant for autologous and allogeneic transplant recipients, an important indicator of regimen-related toxicity. For patients with myeloma undergoing autologous HSCT, 13 of 15 patients relapsed or experienced disease progression, mean time to progression 41 months (range 1.5– 245 months); one patient remains in long-term complete remissions (CR) 20 years post-transplant and another proceeded to receive a sibling allogeneic transplant. All 14 patients who received allogeneic HSCT achieved CR with donor chimerism of >97% at D + 30. There were no cases of grade ≥ 3 acute graft versus host disease (GvHD) and no cases of chronic GvHD. Outcomes are detailed in Table 4.

**Table 4:** Outcomes post-transplant.

Age yrs^a^	Sex	Diagnosis	Type of transplant	Disease status at transplant	Disease status post-transplant	Outcome	Time to relapse or progression months
45	M	Myeloma	Autologous (1st)	PR	PR	Relapse	27
55	M	Myeloma	Autologous (1st)	PR	PR	Relapse	18
58	M	Myeloma	Autologous (1st)	PR	PR	Relapse	1.5
65	F	Myeloma	Autologous (1st)	PR	PR	Relapse	11
55	F	Myeloma	Autologous (1st)	CR	CR	Relapse	18
48	M	Myeloma	Autologous (2nd)	PR	PR	Relapse	4
58	M	AML M6	Allogeneic (sib)	CR2	CR	Alive in CR	–
20	F	AML M5a *t*(9; 11)	Allogeneic (sib)	CR1	CR	Alive in CR	–
43	M	Myeloma	Autologous (1st)	PR	CR	Relapse	15
59	F	Myeloma	Autologous (1st)	CR	CR	Alive in CR	–
66	M	Myeloma	Autologous (1st)	PR	CR	Relapse	19
56	M	Myeloma	Allogeneic (sib)	PR	CR	Alive in CR	–
56	M	Myeloma	Allogeneic (sib)	CR	CR	Alive in CR	–^a^
53	M	Myeloma	Autologous (1st)	CR	CR	Alive post alloHSCT	–
62	M	Myeloma	Autologous (1st)	PR	CR	Relapse	72
65	M	Myeloma	Autologous (1st)	PR	CR	Relapse	45
65	M	Myeloma	Autologous (1st)	PR	CR	Relapse	48
68	M	Myeloma	Autologous (1st)	PR	CR	Relapse	57
60	M	Myeloma	Autologous (1st)	PR	PR	Relapse	7
62	M	CML-CP	Allogeneic (sib)	HR	Mol CR	Alive in mol CR	–
61	M	CML-AP	Allogeneic (VUD)	CP2	Mol CR	Died in CR, PJP	63
61	M	AML M4, FLT3 mutation. Primary refractory.	Allogeneic (VUD)	CR1	CR	Alive in CR	–
54	M	Myeloma, Lambda LCD	Allogeneic (sib)	vgPR	CR	Alive in CR	–
24	M	Secondary AML,	Allogeneic (VUD)	CR1	CR	Alive in CR	–
		(previous HD. Mediastinal radiotherapy, autoHSCT)					
55	M	Secondary AML	Allogeneic (sib)	CR1	CR	Alive in CR	–
45	M	Relapsed myeloma IgA kappa	Allogeneic (VUD)	CR3	CR	Alive in CR	–^a^
57	M	Myeloma	Allogeneic (sib)	vgPR	CR	Alive in CR	–
		IgG kappa					
57	M	Relapsed AML M4	Allogeneic (VUD)	CR2	CR	Died, relapse	5
36	F	AML M7	Allogeneic (sib)	CR1	CR	Died, CNS relapse	10

*PJP* pneumocystis jiroveci pneumonia.

^a^Two myeloma patients relapsed with localised plasmacytomas treated with external beam radiotherapy achieving further CR.

## Discussion

In this study, we demonstrated that ^90^Y-anti-CD66 consistently delivered radiation to red marrow and spleen with minimal uptake by non-haematopoietic organs. The excellent biodistribution of the radiolabelled antibody allowed the use of a simple method based on lean body weight to determine the total activity of ^90^Y for infusion. It should be noted that patients were in remission or very good partial remission at the time of imaging and therapy which would have contributed to the consistent BM targeting as the target antigen CD66 is predominantly expressed on normal myeloid cells. Radiolabelling episodes were reproducible and of high efficiency, both ^111^In and ^90^Y routinely achieving >95% radiochemical purity (RCP) contributing to the observed excellent inter-patient biodistribution and red marrow dosimetry. Similar excellent RCP results have been obtained with besilesomab conjugated to other backbone-substituted derivatives of DTPA such as MX-DTPA [35] and CHXA”-DTPA [49]. At the highest infused activity level of 37.5 MBq/kg, the mean estimated radiation dose to red marrow was 24.5 ± 5.6 Gy, 19.1 ± 8.0 Gy to spleen, 5.8 ± 2.7 Gy to liver, 2.1 ± 0.8 Gy to kidneys, and 2.2 ± 0.9 Gy to lungs with a whole-body dose of 0.9 ± 0.2 Gy. The use of ^90^Y, which emits a high energy (2.3 MeV) beta particle without gamma-emission, allowed patients to receive therapy as outpatients, hospital admission was required only for the completion of standard conditioning. In the period between ^90^Y-anti-CD66 therapy and the start of standard conditioning, there were no serious adverse events attributed to the radiolabelled antibody. Overall toxicities for the transplant period were within the limits anticipated for allogeneic transplantation using fludarabine and melphalan 140 mg/m^2^ or for autologous transplantation with melphalan 200 mg/m^2^. Mucosal toxicity was within anticipated limits with no severe or life-threatening events. However, in patients receiving 200 mg/m^2^ melphalan prior to autologous HSCT for myeloma, there was a trend to grade $\ge$3 dysphagia at the higher activity level as indicated by higher pain scores. Therefore, 37.5 MBq/kg LBW was selected as the activity for a subsequent randomised Phase IIb trial in myeloma autologous HSCT (manuscript in preparation). Although 13 of 15 patients that received autologous HSCT for myeloma experienced disease progression there was a trend for longer time to disease progression between the lowest activity level to the highest with a mean of 14.4 months in cohort 1 and 43.2 months in cohort 4.

Of the 14 patients receiving allogeneic transplants, all engrafted and achieved >97% donor chimerism at day 30 that was maintained. All achieved sustained CRs with ten patients remaining alive in CR, median follow-up of 220 months.

While not encountered in the study, it is possible that the marrow will be the dose-limiting organ because of potential damage to stromal cellular components, compromising stem cell survival. In a canine model where bone-seeking ^166^Ho-EDTMP was used to indirectly target the marrow, the DLT was reversible marrow fibrosis [50]. External beam radiation of 20 Gy or more was shown to cause marrow fibrosis in a rat femur model with >40 Gy causing permanent aplasia [51, 52]. In a clinical trial using an ^131^I-labelled anti-CD45, graft failure occurred in one patient who received a total estimated radiation dose of 42.7 Gy consisting of 12 Gy TBI and 30.7 Gy from MRT [28]. Based on this limited experience, it would seem prudent in future clinical studies to use an upper limit of radiation delivered by targeted radiotherapy to the marrow in the order of 45 Gy.

We were able to demonstrate a dose–response relationship between the infused radiation activity and the effect of this red marrow-targeted radiation on the peripheral blood counts over a 12-day period. In the first activity cohort (5 MBq/kg), two patients showed a modest fall in leucocytes. In contrast, at the highest activity level, all patients had significant falls in leucocytes with the majority of patients having zero neutrophils at the time of admission for standard chemotherapy, demonstrating that the targeted molecular radiotherapy was functioning as a form of ‘pre-conditioning’ rendering the patients aplastic prior to the start of standard conditioning.

The favourable biodistribution and delivery of high absorbed radiation doses targeted to red marrow and spleen with the radiolabelled anti-CD66 mAb added substantial therapy to standard conditioning without additional toxicity and warrants further investigation in Phase II trials, particularly in patients with a high risk of disease relapse post-transplantation.

## Supplementary information

Supplementary Information

## Data Availability

The data that support the findings of this study are available from Telix Pharmaceuticals, but restrictions apply to the availability of these data, which were used under license for the current study, and so are not publicly available. Data are, however, available from the authors upon reasonable request and with permission of Telix Pharmaceuticals.
