# Correction: Efficient bone marrow irradiation and low uptake by non-haematological organs with an yttrium-90-anti-CD66 antibody prior to haematopoietic stem cell transplantation

**DOI:** 10.1038/s41409-026-02810-7

**Published:** 2026-03-04

**Authors:** Kim Orchard, Jonathan Langford, Matthew Guy, Gemma Lewis, Sofia Michopoulou, Margaret Cooper, Clint Zvavamwe, Deborah Richardson, Valerie Lewington

**Affiliations:** 1 Wessex Blood and Marrow Transplantation Programme, Department of Haematology, University Hospital Southampton NHS Foundation Trust, Southampton, UK; 2 NIHR/CRUK Experimental Cancer Medicine Centre, University of Southampton, Southampton, UK; 3 Department of Medical Physics, University Hospital Southampton NHS Foundation Trust, Southampton, UK; 4 Department of Nuclear Medicine, Bart’s and the London NHS Trust, London, UK; 5 Radiopharmacy, University Hospital Southampton NHS Foundation Trust, Southampton, UK; 6 School of Biomedical Engineering and Imaging Sciences, King’s College London, London, UK; 7 Present Address: PET Imaging Centre Facility, King’s College London, London, UK

**Keywords:** Phase I trials, Molecularly targeted therapy

Correction to: *Bone Marrow Transplantation* 10.1038/s41409-024-02317-z, published online 12 Month 2024

During the production process, Table 2 has been typeset wrongly. The correct Table 2 is shown below.

The original article has been corrected.

**Table 2:** Organ dosimetry.

^90^Y dose MBq/kg	Mean infused activity [^90^Y] MBq	Bone marrow		Liver		Spleen		Renal		Pulmonary		Whole body	
		mGy/MBq	Gy	mGy/MBq	Gy	mGy/MBq	Gy	mGy/MBq	Gy	mGy/MBq	Gy	mGy/MBq	Gy
5	407 ± 132	11.8 ± 1.5	4.8 ± 1.6	3.0 ± 0.8	1.2 ± 0.4	9.9 ± 4.9	3.7 ± 1.6	0.3 ± 0.1	0.1 ± 0.1	1.2 ± 1.1	0.5 ± 0.5	0.4 ± 0.1	0.2 ± 0.1
10	781 ± 171	10.3 ± 4.0	8.5 ± 1.3	2.4 ± 1.0	1.2 ± 0.3	11.0 ± 3.2	7.1 ± 3.3	0.4 ± 0.1	0.3 ± 0.1	0.4 ± 0.1	0.3 ± 0.1	0.4 ± 0.1	0.3 ± 0.0
25	1452 ± 69	10.7 ± 2.0	15.7 ± 3.5	2.4 ± 0.8	3.4 ± 1.0	8.5 ± 5.6	12.4 ± 8.2	0.5 ± 0.4	0.7 ± 0.5	1.1 ± 0.6	1.5 ± 0.9	0.4 ± 0.1	0.6 ± 0.1
37.5	2196 ± 310	11.3 ± 2.6	24.6 ± 5.6	2.7 ± .3	5.8 ± 2.7	8.9 ± 3.9	19.1 ± 8.1	1.0 ± 0.6	2.1 ± 1.1	1.0 ± 0.5	2.2 ± 0.9	0.4 ± 0.1	0.9 ± 0.2
Overall mean mGy/MBq		10.1 ± 3.4	--	2.4 ± 1.1	--	9.0 ± 4.0	--	0.5 ± 0.4	--	0.7 ± 0.5	--	0.4 ± 0.1	--

Calculated absorbed radiation dose to critical organs expressed as mGy per infused MBq of 90Y-anti-CD66 and the mean estimated organ absorbed radiation doses in Gray (Gy) with standard deviation.

