# Supplementary material for: Efficient bone marrow irradiation and low uptake by non-haematological organs with an yttrium-90-anti-CD66 antibody prior to haematopoietic stem cell transplantation
Source: Bone Marrow Transplant. 2024 Jun 12;59(9):1247–57. doi: 10.1038/s41409-024-02317-z (PMC11368815; doi:10.1038/s41409-024-02317-z)
Supplement: Supplementary file 1 — Supplementary Information [file 41409_2024_2317_MOESM1_ESM.docx]

**Supplementary information**

# Radiolabelling of DTPA-anti-CD66 with ^111^In and ^90^Y

For imaging and dosimetry 1.5mg of DTPA-anti-CD66 incubated with 200MBq of ^111^In in 0.1 molar ammonium acetate buffer pH 6.0 for 30 minutes at room temperature in a total volume of approximately 1ml. A sample was removed and analysed by high pressure liquid chromatography (HPLC, Varian Prostar) using a Phenomenex BioSep-S 3000 PEEK 300 x 7.5 mm size exclusion column, an inline Bioscan radionuclide detector with 0.1M phosphate buffer containing 0.01M EDTA as the running buffer. In addition thin layer chromatography (TLC) using as stationary phase Tec Control ITLC strips (Biodex, New York), mobile phase 0.1M phosphate buffer with 0.01M EDTA. A labelling efficiency of >95% by both methods was required to permit infusion of the labelled antibody. At the end of the incubation period the binding mixture was diluted to 8ml with normal saline and the volume required to deliver 185MBq was calculated from the measured activity in the syringe. For labelling with ^90^Y a combination of 2M sodium acetate buffer pH 6.0 and 0.1M ammonium acetate buffer pH 6.0 was used, the quantity of ^90^Y depending upon the infused activity level and patient body weight and ranged from 275 - 3000MBq. The labelling mix was incubated for 30 minutes and samples removed for analysis by HPLC and TLC. The labelling mixture was diluted in normal saline prior to infusion of the volume calculated to deliver the required dose depending on treatment cohort and patient body weight.

# Infusion of radiolabelled anti-CD66

All patients were screened for the presence of human anti-murine antibodies (HAMA) in their serum prior to trial entry (Immunomedics, NJ, USA). Subjects received premedication with 10mg chlorpheniramine and 1g paracetamol 30-60 minutes before infusion of radiolabelled antibody. Infusions were performed in a dedicated radionuclide room in the department of Nuclear Medicine, Southampton General Hospital. Infusions were performed through a peripheral vein using a temporary cannula. Blood pressure, pulse rate, oxygen saturations and oral temperature were monitored every 5 minutes during infusion and for 30 minutes post infusion. Infusion of radiolabelled antibody was completed within 15 minutes.

**Patient Specific Dosimetry**

^90^Y has a physical half-life of 64.1 hours and no gamma emissions. With no gamma emissions, imaging can either be carried out directly (using Bremsstrahlung or PET imaging) or indirectly by imaging a tracer administration of ^111^In labelled antibody. For this study, patient-specific dosimetry was estimated using tracer ^111^In imaging and blood sampling enabling pre-therapy dosimetry, rather than direct estimation from the ^90^Y administration.

Basic internal dosimetry can be performed using the methods devised by the Medical Internal Radiation Dose (MIRD) Committee ([18](#_ENREF_18)). The ^90^Y S-values (dose factors - S_k←h_, representing the mean dose to target *k* from source *h*) used in the MIRD calculations were taken from Stabin and Siegel ([53](#_ENREF_53)). Standard Reference Man and Woman organ masses were taken from MIRDOSE 3.1 ([54](#_ENREF_54)) (which utilises amended versions of those found in ICRP Publication 23 [Snyder et al 1975b]), following work done by Cristy and Eckerman [1987] and Stabin et al [1995]). Total bone marrow dose can be derived from the sum of several contributing factors, following the recommendations of the EANM Dosimetry Committee for bone marrow and whole-body dosimetry([17](#_ENREF_17)) for monoclonal antibodies. However, in this case, only the self-irradiating contribution from the bone marrow cells was used, as disease is not expected in the other potential contributing compartments and the contribution from free ^90^Y in the bone was found to be negligible in the Phase I data. All dosimetry measurements were compiled using a MS Excel spreadsheet and validated using independently produced tools.

The infusion of 185MBq ^111^In-labelled anti-CD66 mAb was performed 3-4 weeks before the scheduled transplant date. For the blood activity curve whole blood samples were taken pre-infusion, immediately at the end of infusion (T=0), at 1, 2, 3, 4 hours and then up to 4 further samples taken between days 1 and 7 post infusion. The absolute activity in each sample was determined by counting three 1ml aliquots and a known ^111^In standard in a gamma-counter (Gamma Spectrometer Cobra II, Packard). Circulating total blood volumes for each patient were derived from standard tables ([55](#_ENREF_55)). Typically two washout phases were fitted to the sample curves. The mean urine excretion of ^111^In activity was 24% of injected dose (range 15-40%) over a 7-day period post infusion.

A bone marrow biopsy was taken 24-48 hours post infusion (pi) and the cortical bone removed. The remaining trabecular bone containing red marrow was fixed in formaldehyde, decalcified and sectioned according to standard histological techniques. The cellularity of the biopsy was determined by a trained haematologist and recorded. The percentage cellularity was used to modify the red marrow activity curve as described by Matthews ([3](#_ENREF_3)) using the actual cellularity as a correction factor. In the first 10 patients urine was collected for 7 days post infusion.

*Anti-CD66 ELISA assay*

The concentration of the murine anti-CD66 mAb was measured in serum samples taken post infusion using a validated ELISA([56](#_ENREF_56)). The concentration of anti-CD66 mAb over time followed a biphasic curve similar to the time-activity curve for ^111^In activity with similar T_½_ alpha and T_½_ beta results.

*Gamma-Camera imaging:*

Organ biodistribution of infused ^111^In-labelled MAb was determined from serial whole body gamma-images taken on the day of infusion (day 1) within 1 hour of completion of infusion and at least 3 additional whole-body and SPECT(-CT) acquisitions between days 2 and 7. In all cases, images were acquired using Medium Energy General Purpose (MEGP) collimation on one of two gamma cameras (ADAC Genesys dual-head SPECT camera or Infinia Hawkeye SPECT/CT camera). A photopeak window at 171keV ± 10% was acquired with 2 scatter windows at 209keV ± 6% and 245 ± 4%. Gamma camera calibration was verified for each patient using a standard with a known activity of ^111^In acquired at each whole-body imaging point.

Serial whole body scans were used to estimate activity in the whole-body as a function of time. As the first acquisition takes place before excretion of activity, this can be used to normalise counts per activity based on the decay corrected activity administered. Scatter correction and Geometric Mean calculations were applied. The consistency of the camera’s sensitivity at each time point was validated by imaging a standard within the FoV. Sufficient counts were acquired to cap noise over the standard sample region to less than 1%. Consistency was assessed by verifying that the calculated half-life of the ^111^In sample was within 10% of the actual physical half-life over all time points. Mean whole-body dose and retention was calculated from this data using the MIRD schema, as detailed above.

In the last 14 patients of the study, additional scans were obtained using SPECT/CT (Infinia Hawkeye, GE Healthcare, USA) to allow accurate absolute organ activities to be determined. Scatter and attenuation correction were applied during the OSEM reconstruction. Estimated mean absorbed radiation dose for the bone marrow, liver, spleen, kidneys and lungs were derived using the MIRD schema.

Retention of ^111^In in the lumbar spine was used as a surrogate of actual bone marrow uptake. Allowance was made for the impact of the Partial Volume Effect (PVE) in the L2, L3, L4 and L5 vertebrae.

**Transplant conditioning regimens post MRT.**

The ^90^Y-anti-CD66 was given on D-14 before the day of transplant on D0 in all patients. All patients in the study were treated as day cases and discharged after the infusion of ^90^Y-anti-CD66. Patients receiving autologous transplants for myeloma were reviewed as on out-patient 7 or 8 days post infusion for medical review and subsequently admitted to the transplant unit on D-2 to receive intravenous melphalan 200mg/m^2^ as per standard autologous HSCT. Patients undergoing allogeneic HSCT were admitted on D-10 prior to the day of transplant, 5 days after the infusion of ^90^Y-anti-CD66, to start standard ‘FMC’ conditioning with intravenous fludarabine 30mg/m^2^ D –7 to –3 inclusive, melphalan 140mg/m^2^ D –2 and the anti-CD52 monoclonal antibody alemtuzumab, total dose 30mg for sibling transplants and 50mg for unrelated donor transplants. Donors and recipients were tissue-typed using high resolution DNA typing, full 10/10 or 1 antigen mismatched unrelated donors were permitted except DRB1 mismatches. GvHD prophylaxis consisted of ciclosporin A and methotrexate. In the absence of active GvHD ciclosporin A was tapered from day + 60 and withdrawn by day 100-120. Prophylactic antimicrobial agents were used as per centre protocols. Chimerism was performed from D +30 and subsequently every 30 days using three informative polymorphisms between recipient and donor by SNP analysis.
